# A Review of the Dietary Intake, Bioavailability and Health Benefits of Ellagic Acid (EA) with a Primary Focus on Its Anti-Cancer Properties

**DOI:** 10.7759/cureus.43156

**Published:** 2023-08-08

**Authors:** Philip Harper

**Affiliations:** 1 Life Sciences, University of Southampton, Southampton, GBR

**Keywords:** antimicrobial, cancer, anti-inflammatory, antioxidant, polyphenol, ellagitannin, ellagic acid

## Abstract

Ellagitannins (ET) and ellagic acid (EA) are polyphenols, present in common foods, which may exhibit significant health benefits against inflammation, infection and cancer. EA is metabolised by the gut flora to produce urolithins, which are absorbed into the systemic circulation. Urolithins are widely documented to reduce oxidative stress associated with many diseases including cancer, heart disease and liver damage. In particular, Urolithin C and D have been shown to have high anti-oxidant properties through the inhibition of reactive oxygen species (ROS). The anti-inflammatory properties of EA have been demonstrated through the down-regulation of pro-inflammatory enzymes such as COX-2 and iNOS as well as decreasing the expression of adhesion molecules. EA also regulates the gut microflora and possesses antimicrobial activity against various strains of harmful bacteria such as methicillin-resistant Staphylococcus aureus (MRSA) and Helicobacter pylori. Numerous studies have documented the anticarcinogenic benefits of EA and have been performed on, but not limited to, prostate, colon and breast cancer cell lines and in vivo models. Conventional treatments for cancer, such as chemotherapy, can often be associated with significant side effects such as fatigue, hair loss and alopecia. Naturally-occurring food substances such as ETs potentially offer a risk-free preventative measure against cancer and could perhaps be used in synergy with current treatments. More level 1 studies are required to inform the evidence-base on this topic.

## Introduction and background

Polyphenols are a large group of phytochemicals which form part of a plant’s immune system protecting them from bacteria, viruses and fungi. Ellagitannins (ETs) and ellagic acid (EA) are commonly occurring polyphenols present in certain fruits, nuts and seeds including pomegranate, raspberries, strawberries, walnuts and almonds. In recent years, polyphenols have been extensively researched due to their potential health benefits. They represent a major source of antioxidant in our diet and have been shown to have beneficial health effects in cancer, cardiovascular disease, inflammation, diabetes and Alzheimer’s disease [1]. 

This review explores the bioavailability, metabolism and health effects of ET and EA with a primary focus on their anti-cancer properties. A narrative review of the literature was conducted using PubMed, Web of Science, Scopus and reference lists of chosen articles. Keywords including ‘polyphenol’, ‘ellagitannins’, ‘anti-inflammatory’, ‘anti-oxidant’ and ‘cancer’ were used to narrow the search. Only English-written papers were included and the full text of each paper was reviewed prior to its inclusion in this review. Recently published articles were prioritized. References were managed using Endnote software.

## Review

Dietary intake

Various studies have attempted to estimate the average dietary intake of polyphenols and ETs. In a Spanish diet, the mean daily intake of total polyphenols was estimated between 2,590 and 3,016 mg/day [2]. France and Denmark produced similar results of 1,193 ± 510 mg/day and 1,786 mg/day, respectively. Finland and Greece had more modest estimations with average daily intakes of 863 ± 415 mg/day and 744 mg/day, respectively. The daily intake of ET was 12 mg/day in Finland, 13 mg/day in America and only 5 mg/day in Germany [3]. In France, the average person consumes around 1.7 kg of strawberries every year, which is their primary source of ETs. This would correspond to a daily consumption of around 0.4 mg/day total EA [4]. As demonstrated by these studies, polyphenol and ET intake varies drastically between countries. Accurately measuring the daily intake of polyphenols is extremely challenging due to the inability to control all aspects of a subject’s diet. 

Bioavailability and metabolism

EA can occur in either its free form, EA glycosides, or bound as ETs. ETs are hydrolysed to EAs under physiological conditions and then metabolised by the intestinal gut flora to produce urolithins, which are absorbed into the circulation. The low bioavailability of ETs are likely due to their large size and high polarity with the presence of C-C linkage. ETs which are sensitive to hydrolysis in the stomach and duodenum release EAs which are also poorly bioavailable due to low water solubility, extensive degradation before absorption and its irreversible binding to DNA, which may affect transcellular absorption [5]. When mice were given ETs (from raspberries or pomegranates) 10% of the total EA dose was detected in the urine and faeces. However, there was virtually no trace of it in the blood or tissues [6]. In a human subject, EA was detected in plasma at a maximum concentration after one hour of ingesting pomegranate juice but was rapidly eliminated after four hours [7].

Studies have shown that urolithins are much more readily bioavailable and appear in human systemic circulation within a few hours of pomegranate ingestion reaching maximum concentrations between 24 and 48 hours. They were found to exist in both free and conjugated forms in the plasma and urine [8,9]. In view of this they are considered the bioactive forms of ETs.

Toxicity of ET

Before delving into the potential health benefits of ETs and EA, one must consider the negative effects associated with their intake. Tasaki et al. performed a toxicity study on EA using F344 rats with doses ranging between 9.4 and 42.3 g/kg of body weight. Haematology and serum biochemistry revealed sporadic alterations in mean corpuscular volume (MCV), aspartate transaminase (AST) and ALP observed in both genders but this was not felt to be treatment-related. Histological testing revealed sporadic lesions in the lungs, heart, liver and kidneys. However, incidence of lesions was similar to the control group. Body weight gain was slightly decreased in the female group but not to the extent of affecting biochemical markers. In conclusion, the no-observed-adverse-effect level in female rats was estimated to be 3,254 mg/kg/day, significantly higher than normal dietary amounts [10].

Polyphenols bind iron in the intestines and can prevent it from being absorbed. This can have a beneficial impact in cases of iron overload and preventing formation of oxygen free radicals which will be discussed later [11]. However, it can also be detrimental and may increase the risk of iron-deficient anaemia.

Binding of polyphenols to digestive enzymes such as amylase, protease and lipase can impair their function and disturb the function of the gastrointestinal system. This could be particularly harmful to people with food intolerances who lack certain enzymes such as gluten and lactose intolerance, coeliac disease or cystic fibrosis. Further to this, elderly people are at higher risk of digestive enzyme deficiency and ingestion of a high polyphenol diet could exacerbate this [12]. Other negative impacts include disturbing the gut microbiome and impacting the metabolism of certain drugs by either increasing or diminishing their therapeutic effect.

Antioxidant effects

The cellular injury caused by oxidative stress and free radicals has been associated with many diseases including cancer, heart disease and liver damage. Polyphenols are widely considered to have strong anti-oxidant potency acting as reactive oxygen species (ROS) scavengers (Type 1 antioxidants), peroxide decomposers and electron donors (type 2 antioxidants) [13]. EA exerts its effects against free radicals using type 1 and type 2 mechanisms. Importantly, it inhibits the endogenous production of radial hydroxyl which is responsible for tissue and DNA damage. It also chelates and subtracts metal ions such as Fe2+ and copper ions involved in the production of free radicals [14]. As previously mentioned, only trace amounts of EA have ever been found in human blood whereas their metabolites, urolithins, are readily absorbed into the systemic circulation.

Urolithin A, when tested as a direct radical scavenger showed an IC50 OF 152.66mM in the 2,2-diphenyl-1-picrylhydrazyl (DPPH) test compared to EA which had an IC50 of 6.6 mM. However, rather than being prolific free radical scavengers Urolithin A exerted most of its antioxidant effects through the inhibition of oxidase enzymes, such as MAO-A and tyrosinase, with IC50 values of 71.44 and 29.4 mM, respectively [15]. Bialonska et al. demonstrated that Urolithin C and D exerted the highest antioxidant activity with IC50 values of 0.16 and 0.33 μM, respectively, when compared to IC50 value of 1.1 of the EA. Urolithin C had superior lipophilicity and hence was more bioavailable that Urolithin D [16].

Anti-inflammatory effects

The effects of pomegranate extract (POMx) have been tested on collagen-induced arthritis (CIA) in mice. POMx is a highly rich source of ETs. It was found that consumption of POMx delayed the onset and reduced the incidence of CIA at doses of 13.6 mg/kg and 34 mg/kg. Activated macrophages produce several inflammatory mediators and infiltrate joints in arthritis. One such mediator is nitric oxide (NO). POMx was shown to significantly inhibit NO production in LPS-stimulated macrophages at a dose of 20 µg/ml. The POMx-fed mice demonstrated reduced joint infiltration by the inflammatory cells and levels of interleukin-6 (IL-6) were significantly decreased [17].

Endothelial cell expression of adhesion molecules has been recognized as an early step in inflammation. Papoutsi et al examined the effect of EA from walnuts on the expression of vascular cell adhesion molecule (VCAM)-1 and intracellular adhesion molecule (ICAM)-1 in human aortic endothelial cells. Results showed that EA significantly decreased the TNF-α-induced endothelial expression of both VCAM-1 and ICAM-1 at a concentration range of 0·1-10 μm [18]. Mirzaie et al. conducted a randomised controlled trial where individuals with Inflammatory Bowel Disease (IBS) received 180mg of EA per day for two months. They found a significant reduction in the IBS severity score in the treatment group owing to less severe abdominal pain/distension and frequency of symptoms [19].

Anti-microbial and probiotic effects

It has been shown that EA can influence the gut microflora by either destroying harmful bacteria or enhancing the growth of beneficial bacteria. When EA-rich foods, such as pomegranate, are consumed the ETs accumulate in the large intestines, where they interact with and promote the gut microflora. Using fluorescence in situ hybridization (FISH) analysis, POMx enhanced the growth of total bacteria, bifidobacterium spp and Lactobacillus spp using human faecal samples. These two groups of bacteria are associated with many health benefits. POMx did not influence the Clostridium coccoides-Eubacterium rectale group and the C. histolyticum group. The lack of growth enhancement in the latter group has clinical significance as it contains proteolytic bacteria, the metabolism of which have been linked to IBS and colorectal cancer [20].

EA along with other pomegranate-containing compounds have antimicrobial activity when assayed against various strains of bacteria including E. coli and methicillin-resistant Staphylococcus aureus (MRSA) [21]. They elicited a dose-dependent bactericidal effect in Helicobacter pylori by inhibiting the action of Arylamine N-acetyltransferase and Staph. aureus with an IC50 value of 0.47 mM through the inhibition of N-acetylation of 2-aminofluorene [22,23]. They are believed to disrupt the cell membrane in both gram positive and gram negative bacteria through direct binding or binding with biomolecules such as peptidoglycans in cell membranes [24]. Mycobacterium abscessus (Mabs) is a cause of various types of infection including respiratory infection in patients with cystic fibrosis. EA demonstrated antimicrobial activity against Mabs due to its high binding affinity to Dihydrofolate reductase at a minimum inhibitory concentration of 1.56 mg/mL and bactericidal concentration of of 3.12 mg/mL [25].

Unfortunately, the inhibitory effect of polyphenols is not just limited to pathogens. The inhibitory effect of a variety of polyphenols on the gut microbiome is noted in multiple studies including myricetin inhibiting the effect of intestinal lactic acid bacteria, epigallocatechin inhibiting non-pathogenic E.coli and quercetin inhibiting Ruminococcus gauvreauii [26-28].

Anti-cancer effects

Polyphenols mediate their anti-cancer effects through antioxidant, anti-inflammatory and anti-proliferative mechanisms as well as influencing subcellular signalling pathways and inducing cell-cycle arrest and apoptosis. Many different types of cancer have been investigated in vitro. This review will focus on prostate, colon and breast cancer.

Prostate Cancer

EA treatment of prostate cancer PC3 cells resulted in decreased cancer cell proliferation through a reduction of phosphorylated STAT3, ERK and AKT signalling proteins after 72 hours in a dose-dependent manner (0-100 µM) [29]. Other studies have reached similar conclusions by assessing different components of pomegranate such as the peel, juice seeds and seed oil (rich sources of ETs) on various prostate cancer cell lines. They consistently demonstrated pro-apoptotic and inhibitory effects on cancer cell proliferation. In vivo they demonstrated potent inhibition of PC3 xenograft growth in mice [30,31]. Urolithins have also been shown to inhibit the growth of both androgen-dependent and androgen-independent prostate cancer cell lines in vitro with IC50 values lower than EA, however, the exact mechanisms by which they exert these effects need to be investigated [32]. Furthermore, the IC50 values of urolitihins were higher than what is physiologically achievable.

Inflammation is a hallmark of many cancers, in particular prostate cancer, and is dependent on the transcription factor NF-kB. NF-kB activation leads to immune activity, inflammation and cell proliferation whilst also upregulating the transcription genes that produce collagenases, cell adhesion molecules and inflammatory cytokines. It is also associated with the transcription of genes involved in cell survival and inhibiting apoptosis. Inflammation can result in persistent oxidative stress in cancer cells and ROS may give these cancer cells a survival advantage. EA has been shown to prevent prostate cancer growth, by inhibiting inflammatory pathways including the NF-kB pathway [32].

Another hallmark of cancer is angiogenesis. Sartipour et al. administered oral POMx to severe combined immunodeficient mice which had been previously injected with LAPC4 human prostate cancer cells. After four weeks of treatment POMx decreased prostate cancer xenograft size, tumour vessel density, vascular endothelial growth factor (VEGF) levels and HIF-1 alpha expression, thereby suggesting that POMx has an inhibitory effect on tumour-associated angiogenesis [33].

A clinical trial was carried out which looked at the effects of pomegranate juice on rising prostate specific antigen (PSA) in prostate cancer patients after surgery or radiotherapy was conducted. The mean length of time for PSA to double was significantly prolonged following daily pomegranate juice consumption [34].

Colon Cancer

EA was extracted from the pulp and seeds of five different types of raspberries and exerted antiproliferative effects against human colon carcinoma cells in vitro. The extent of this effect was correlated with the content of EA in the extract [35]. The activation of Wnt signalling pathways are known to play a key role in human colon carcinogenesis. Using the 293T colon cancer cell line it was observed that Urolithin A inhibited the Wnt signalling pathway at physiologically relevant concentrations - IC50 of 39 μM [36]. The doses of ET and EA needed to inhibit Wnt signalling were unfortunately not physiologically relevant.

Cytochrome p450-1 (CYP1) enzymes are involved in the activation of procarcinogens into cancer-inducing chemicals. ET and urolithins showed a significant dose-dependent inhibition of CYP1 enzymes in HT-29 colon cancer cells, whilst also demonstrating anti-proliferative effects mediated through cell cycle arrest in the G0/G1 and G2/M stages followed by induction of apoptosis [37]. González-Sarrías et al. reached similar conclusions and found that EA, Urolithin A and Urolithin B modulated phase I and phase II detoxifying enzymes in colon cancer Caco-2 cells. The three compounds induced the expression and activity of CYP1A1 which, unlike the other CYP enzymes, is involved in detoxification [38].

5-Fluorouracil (5-FU) remains a first line treatment for colorectal cancer. However, only 10-15% of patients with advanced colorectal cancer respond positively to 5-FU monotherapy. Therefore, new strategies to enhance the 5-FU effectiveness are critically needed. The co-treatment of 5-FU with Urolithin A, resulted in decreased IC50 values for 5-FU and cell cycle arrest at the G2/M phase together with a slight increase in caspases 8 and 9 activation. Urolithin A may therefore potentiate the effects of 5-FU on colon cancer cells. This potentially means that lower doses of 5-FU could be used to achieve therapeutic effects thereby decreasing the likelihood of adverse effects [39].

Black raspberries have a high concentration of phenolic compounds including ETs and flavonoids. A clinical trial examined the effects of concentrated berry power in 20 colorectal cancer (20g freeze dried berry powder diluted in 100ml of water consumed x3 times a day for 1-9 weeks). Immunohistochemistry demonstrated that blackberries protectively modulated a number of anti-cancer proteins including β-catenin (Wnt pathway), Ki-67 (cell proliferation), terminal deoxynucleotidyl transferase dUTP nick end labeling (TUNEL) (apoptosis) and CD105 (angiogenesis) [40]. Smad4 is a tumour suppressor gene, which, when poorly expressed, is associated with worse outcomes in colorectal cancer. Colorectal specimens from patients who had ingested black raspberries showed increased Smad4 expression in the epithelium compared to control subjects [41]. Upregulating Smad4 expression in the colonic epithelium could reduce the risk of malignant transformation in colonic tumours. Of course, a limitation with foods such as blackberries is the varying concentrations of phenolic compounds within them, which thereby lessens the reliability and consistency of chemopreventive effects.

Breast Cancer

Polyphenols from fermented juice, pericarp and oil inhibited the growth of breast cancer cell lines in vitro. This effect was greater in the oestrogen-dependent MCF-7 line than the oestrogen-independent MB-MDA-231 line. Fermented pomegranate juice polyphenols consistently showed about twice the anti-proliferative effect as fresh pomegranate juice polyphenols. Oestrogen synthesis was inhibited by a 60-80% reduction in aromatase activity.

As previously mentioned, a tumour needs to receive sufficient nutrients and oxygen and have the ability to evacuate waste in order to sustain growth. It achieves this through angiogenesis and VEGF is the most potent stimulator of this process. EA markedly inhibits angiogenesis-associated activities including proliferation, migration/invasion and capillary formation on VEGF-stimulated endothelial cells in vitro at doses of 2.5 to 20 μM. It directly inhibited VEGFR-2 tyrosine kinase activity and its downstream signalling pathways including MAPK and PI3K/Akt in endothelial cells. In vivo, EA inhibited vessel formation in chick chorioallantoic membrane and sprouts formation of chicken aorta. In breast cancer xenografts, EA significantly inhibited MDA-MB-231 cancer growth and P-VEGFR2 expression. The authors concluded very low toxicity of EA given there were no abnormal morphological changes to the cancer cell lines and in vivo there was sprout recurrence from the chick aortic arch once EA was removed [42]. To our knowledge, there have been no clinical studies performed regarding the effects of EA on breast cancer.

Synergistic Effects of Polyphenols

EA is widely considered the key compound in pomegranate in terms of its anti-carcinogenic effects. However, it is believed that its action is enhanced by other polyphenols present in pomegranate. Seeram et al. compared the effect of pure pomegranate juice with individual polyphenols (EA and punicalagin) on human oral, prostate and colon cancer cells. It found that the polyphenols independently decreased the viable cell number of cancer cells but not nearly as much as the pure pomegranate juice. The juice also had a more profound effect on inducing apoptosis compared with the separated polyphenols [43].

## Conclusions

In recent years, an abundance of research has led to the idea that ETs, EA and their derived metabolites may be protective against many diseases including cancer. The prospect of natural chemotherapy is very appealing because medical chemotherapy often produces distressing side-effects, which may hinder compliance. The in vitro and in vivo evidence of ET health benefits is strong, however, there is a dearth of research assessing the effects of ETs in human subjects. The clinical significance of many of the aforementioned studies isn’t clear as ET are not readily found in the bloodstream following ingestion of ET-rich foods. Studies involving EA and/or urolithins are perhaps more clinically revelant as these have better bioavailability. EA and urolithins have been shown to accumulate in the prostate and intestines where they may exert their most potent anti-cancer effects. It is unclear why they localise here in relation to other organs. 

Clinical studies investigating the timing of ET-rich supplements in cancer therapy would be useful. For example, neo-adjuvant versus adjuvant treatment and in combination with other existing therapies such as chemoradiotherapy or hormone therapy. Current evidence on urolithins potentiating the effects of chemotherapy agents such as 5-FU are very promising and could significantly improve the quality of life of patients undergoing chemotherapy treatment. There is also benefit in the cost-effectiveness of chemopreventive foods which may represent a more affordable option for developing countries than pharmacological agents. Randomised controlled trials should be carried out to investigate recurrence rates following primary cancer treatment used in conjunction with ET supplementation. Finally, the therapeutic doses of EA and urolithins need to be better defined in order to analyse the risk-benefit profile of oral supplementation. The negative health impacts of polyphenols and their metabolites have been discussed and should be considered when conducting clinical trials.
